# Localization of beta and high-frequency oscillations within the subthalamic nucleus region

**DOI:** 10.1016/j.nicl.2017.07.018

**Published:** 2017-07-24

**Authors:** B.C.M. van Wijk, A. Pogosyan, M.I. Hariz, H. Akram, T. Foltynie, P. Limousin, A. Horn, S. Ewert, P. Brown, V. Litvak

**Affiliations:** a Department of Neurology, Charité - University Medicine Berlin, Berlin, Germany; b Wellcome Trust Centre for Neuroimaging, UCL Institute of Neurology, London, United Kingdom; c Nuffield Department of Clinical Neuroscience, University of Oxford, John Radcliffe Hospital, Oxford, United Kingdom; d Unit of Functional Neurosurgery, Sobell Department of Motor Neuroscience and Movement Disorders, UCL Institute of Neurology, London, United Kingdom; e Department of Clinical Neuroscience, Umeå University, Umeå, Sweden; f Victor Horsley Department of Neurosurgery, National Hospital for Neurology and Neurosurgery, London, United Kingdom; g Berenson-Allen Center for Non-Invasive Brain Stimulation and Division of Cognitive Neurology, Department of Neurology, Beth Israel Deaconess Medical Center, Harvard Medical School, Boston, MA, USA; h Department of Neurology, Massachusetts General Hospital, Harvard Medical School, Boston, MA, USA; i Medical Research Council Brain Network Dynamics Unit at the University of Oxford, Oxford, United Kingdom

**Keywords:** Electrophysiology, Oscillations, Basal ganglia, Cross-frequency coupling, Parkinson's disease, Motor system

## Abstract

Parkinsonian bradykinesia and rigidity are typically associated with excessive beta band oscillations in the subthalamic nucleus. Recently another spectral peak has been identified that might be implicated in the pathophysiology of the disease: high-frequency oscillations (HFO) within the 150–400 Hz range. Beta-HFO phase-amplitude coupling (PAC) has been found to correlate with severity of motor impairment. However, the neuronal origin of HFO and its usefulness as a potential target for deep brain stimulation remain to be established. For example, it is unclear whether HFO arise from the same neural populations as beta oscillations. We intraoperatively recorded local field potentials from the subthalamic nucleus while advancing DBS electrodes in 2 mm steps from 4 mm above the surgical target point until 2 mm below, resulting in 4 recording sites. Data from 26 nuclei from 14 patients were analysed. For each trajectory, we identified the recording site with the largest spectral peak in the beta range (13–30 Hz), and the largest peak in the HFO range separately. In addition, we identified the recording site with the largest beta-HFO PAC. Recording sites with largest beta power and largest HFO power coincided in 50% of cases. In the other 50%, HFO was more likely to be detected at a more superior recording site in the target area. PAC followed more closely the site with largest HFO (45%) than beta power (27%). HFO are likely to arise from spatially close, but slightly more superior neural populations than beta oscillations. Further work is necessary to determine whether the different activities can help fine-tune deep brain stimulation targeting.

## Highlights

• LFPs were recorded from multiple sites within and around the subthalamic nucleus.
• Sites with largest beta and high-frequency oscillations (HFO) were identified.
• HFO were located slightly more superior than beta oscillations.
• Phase-amplitude coupling more closely followed the site with largest HFO.
• This work hints at different neural generators for beta and HFO.

## 1 Introduction

The subthalamic nucleus (STN) is a prime target for deep brain stimulation (DBS) for Parkinson's disease. Accurate positioning of electrodes is essential for achieving optimal clinical outcome. Although the STN is small in size, it is thought to consist of functional compartments as defined by their anatomical connections to limbic, associative, or sensorimotor cortical and subcortical structures with varying degrees of overlap (Parent and Hazrati, 1995, Lambert et al., 2012, Haynes and Haber, 2013). Indeed, stimulation through electrode contacts placed in the dorsolateral, or superolateral (sensorimotor) portion of the STN has been found to be most effective in reducing motor impairment (Lanotte et al., 2002, Saint-Cyr et al., 2002, Godinho et al., 2006).

Local field potentials recorded post-operatively from these DBS electrodes have revealed that Parkinsonian bradykinesia and rigidity are associated with excessive beta band oscillations (Gatev et al., 2006, Hammond et al., 2007, Oswal et al., 2013). Both dopaminergic medication and DBS reduce beta band amplitude along with improvements in clinical motor scores (Kühn et al., 2006, Kühn et al., 2008, Kühn et al., 2009, Weinberger et al., 2006, Ray et al., 2008, Eusebio et al., 2011). In line with this, the sites where strongest beta band oscillations are recorded during electrode implantation concur with the sites of any clinical stun effect (Chen et al., 2006), and often coincide with the positions of the DBS contacts selected for chronic stimulation (Yoshida et al., 2010, Zaidel et al., 2010).

Recently, high-frequency oscillations (HFO) within the 150–400 Hz range have also been associated with the disease. HFO may show abnormally strong phase-amplitude coupling (PAC) with the beta band that correlates with bradykinesia and rigidity UPDRS scores and is reduced with levodopa treatment (López-Azcárate et al., 2010, Özkurt et al., 2011, van Wijk et al., 2016). Furthermore, its occurrence is indicative of tremor (Hirschmann et al., 2016). This suggests that HFO may be relevant for improving DBS targeting and treatment. At present, little is known about the neuronal origin of HFO and whether it arises from the same neural populations as beta oscillations.

In the current study, we explored whether beta band oscillations and HFO can be localized to the same spatial location within the STN area. This work complements earlier findings by Wang et al. (2014) who found HFO to be localized towards the anterior part of STN using intra-operative microelectrode recordings, and Yang et al. (2014) who localized beta-HFO PAC in the superior part of the STN. Here we explicitly examined the localization of HFO with respect to beta band oscillations to infer whether these can be considered to arise from different neural populations. This is particularly relevant for assessing the potential contribution of HFO power and beta-HFO PAC as independent data features for fine-tuning DBS.

## 2 Methods

All patients in this study were diagnosed with Parkinson's disease according to the Queen Square Brain Bank Criteria (Gibb and Lees, 1988), and underwent surgery at the National Hospital for Neurology and Neurosurgery in London, UK. Intraoperative LFP recordings were obtained from 26 subthalamic nuclei in 14 patients as part of the clinical procedure to aid localization and were provided for analysis by the clinical team in anonymised form. All patients gave their written informed consent to the procedure including LFP recordings. Average age of patients at the time of surgery was 59 years (range 51–65) with a disease duration of 10 years (range 6–17) after diagnosis. Pre-operative Unified Parkinson's Disease Rating Scale (UPDRS) part III scores (motor section) on average totalled 46 points (range 28–72) off medication, with an average sum of hemibody bradykinesia and rigidity items of 12 (range 6–22).

Using a T1 and T2-weighted MRI guided and MRI verified approach, DBS electrodes were implanted bilaterally in the visualized STN as per the centre's standard procedures (Foltynie et al., 2011). The intended target for the lowermost contact of the DBS electrode (‘C0’) was determined on a stereotactic axial T2-weighted MRI scan lying at the level of the largest diameter of the nucleus ruber, which corresponds to the ventral part of the STN (Hariz et al., 2003). The centre of the subthalamic nucleus was identified in a plane 0–1 mm behind the anterior border of the nucleus ruber (Bejjani et al., 2000). A double oblique trajectory was planned to avoid sulci and ventricles. Surgery was performed under local anaesthesia after overnight withdrawal of medication. During the LFP recordings, patients were awake and instructed to keep eyes opened and rest without voluntary movement or speech. Excluding the 4 hemispheres without a clear peak in the HFO range (see below), the lowermost contact was eventually fixed either at the target point location (6 nuclei), 1 mm below (1 nucleus) or 2 mm below (15 nuclei), depending on observed beta activity in the LFP recordings and the patient's response to intraoperative stimulation that took place after the LFP recordings. A fixation site of 2 mm below the surgical target point was often chosen to ensure placement of at least two contacts within the STN.

The DBS leads used for recordings were of model 3389, (Medtronic, Minneapolis, MN, USA), and contained four cylindrical platinum-iridium contacts with a length of 1.5 mm and a diameter of 1.27 mm each and were separated by 0.5 mm. Hence the distance between the centre of adjacent contacts was 2 mm. Monopolar recordings (with linked ear reference) were examined but found to be contaminated by high amplitude alpha activity. An offline bipolar derivation between adjacent contacts eliminated the alpha peak, which is therefore likely to arise through volume conduction. This observation is in accordance with recent experimental work demonstrating superior performance for bipolar compared to monopolar macroelectrode recordings in detecting beta oscillations within the STN (Marmor et al., 2017). We decided to use only the derivation between the two lowermost contacts in this study to limit a reduction in recorded signal induced by the tip of the lead while sliding through the tissue (stun effect). Local field potentials were typically recorded while advancing the leads in 2 mm steps from 4 mm above until 2 or 4 mm below the surgical target point within the STN. For the majority of cases, this resulted in recordings from 4 sites (+ 4, + 2, 0, − 2 mm, for 16 nuclei in 11 patients), while for 5 nuclei (in 3 patients) we had recordings from 5 sites available (+ 4, + 2, 0, − 2, − 4 mm), and in 1 nucleus from 3 sites (+ 4, 0, − 2 mm). This sums up to 92 sampling points. For hemispheres not included in the results (see later) we had either 4 (2 nuclei in 2 patients) or 5 (2 nuclei in 2 patients) recording sites.

**Fig. 1:**
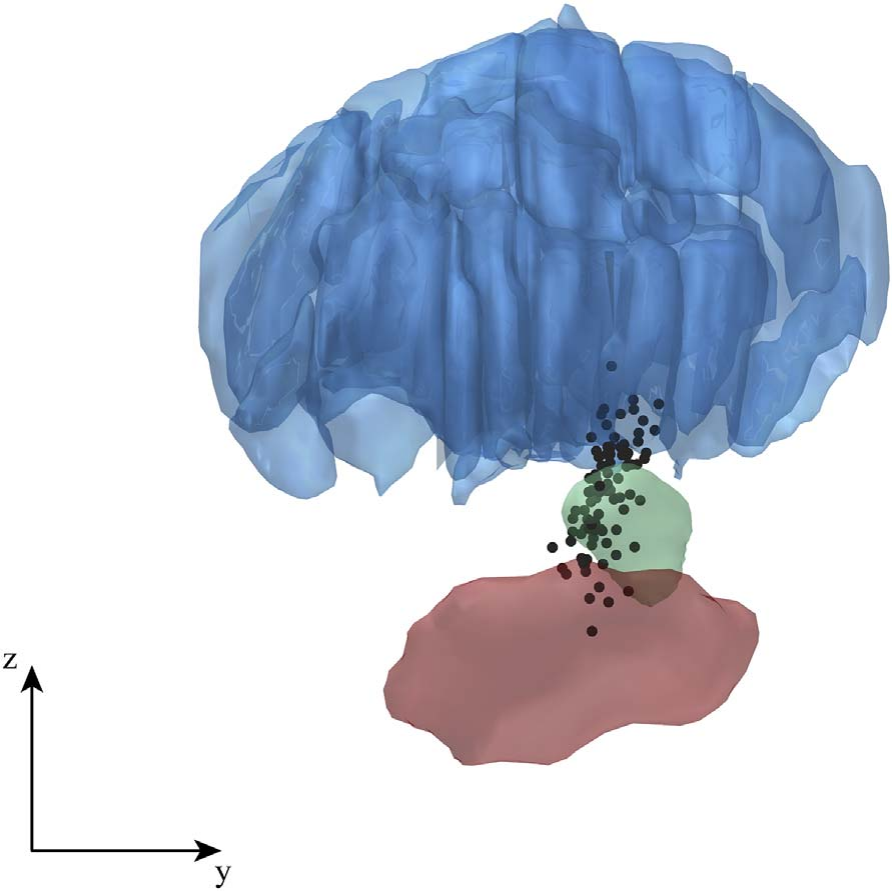
Lateral view of all intra-operative recording sites across patients. Each black dot represents the midpoint between the locations of the two lowermost contacts at each recording site as we used a bipolar derivation. The actual volume from which activity was picked up was much larger than a single point in space. The figure contains recordings from both the right and left hemisphere (mirrored in the y-axis). A relatively wide spatial range was sampled including sites inside the STN (shown in green), and sites where it was more likely to pick up activity from the thalamus (blue) or substantia nigra (red). A total of 92 points are depicted (all cases included in the study). The inserted axes indicate 1 cm in each direction. (For interpretation of the references to color in this figure legend, the reader is referred to the web version of this article.)

We localized all recordings sites in 3D using the Lead-DBS toolbox (Horn and Kühn, 2015) version 1.5.1.2. This involved marking the electrodes’ positions on immediate postoperative stereotactic axial T2-weighted MRI images (voxel size 0.488 × 0.488 × 2 mm), co-registration with the pre-operative T1 scan, and (nonlinear) normalization to MNI space (using DARTEL implemented in SPM12). This allowed us to visualize recording sites of all patients together in one figure (Fig. 1). Left-hemisphere electrodes were flipped to the right hemisphere. We used a 7 T-based atlas to outline the STN and substantia nigra (Wang et al., 2016), and a histological atlas to outline thalamic structures (Chakravarty et al., 2006) warped to the ICBM Nonlinear 2009b template (Ewert et al., 2016). The bipolar recordings were visualized as a single point midway between the location of the two lowermost contacts.

The average duration of a recording per site was 71 s (range 21–94 s). The earliest recordings were collected with a sampling rate of 1600 Hz (2 patients) or 1500 Hz (1 patient) before selecting a fixed sampling rate of 1024 Hz for the remainder of the cohort (11 patients). A low-pass filter was set at half the sampling rate. All subsequent analyses were performed using custom-written Matlab scripts (R2014a, The Mathworks Inc., Natick, USA). We eliminated 50 Hz line noise artefacts and subsequent harmonics up to half the sampling rate using 4th order Butterworth notch filters with cut-off frequencies of ± 1 Hz around the centre frequency. In addition, notch filters with the same settings were used to remove sharp artefacts at other frequencies that might have originated from other devices in the operating theatre. These often occurred for multiple frequencies above 50 Hz without being exact harmonics. They were easily identifiable by the sharpness of their spectral peaks, which was similar to that of the 50 Hz harmonics. By contrast, HFO peaks were much broader, spanning a range of 50 Hz or more.

**Fig. 2:**
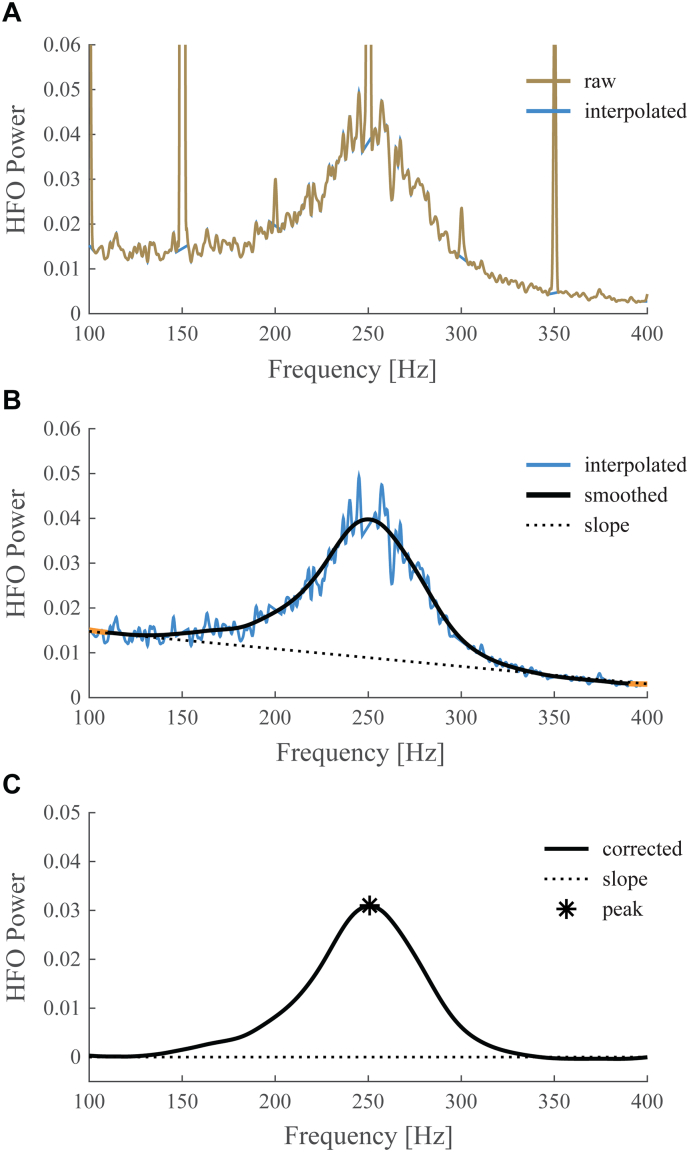
Peak detection in the HFO range. This figure illustrates the procedure we followed for HFO peak detection applied to a case with pronounced 50 Hz harmonics. Firstly, spectral values within 3 Hz around harmonics were removed and interpolated (A). Secondly, the interpolated time series was low-pass filtered and a linear slope spanning the 100–400 Hz range was determined based on the average power within the 100–110 Hz and 390–400 Hz intervals shown in orange (B). The linear slope was then subtracted from the smoothed curve and the maximum value of the resulting curve was detected (C).

For each nucleus, we compared absolute spectral power across sites to identify the site with the largest beta peak and the site with the largest HFO peak. Power spectral densities were computed using Matlab's pwelch method with Hanning windows of 1 s duration and 50% overlap. Spectral dips due to the application of notch filters were masked by removing power values for frequencies ± 3 Hz around the centre frequency and a subsequent linear interpolation of the missing values. All maxima within the beta band interval (13–30 Hz) were detected using the first and second derivatives of the spectra. The maximum with the largest value was designated as the largest peak, and was compared against those obtained from other recording sites. The same procedure was followed for the detection of the largest HFO peak (within 150–400 Hz), except that the power spectrum itself was first smoothed across frequencies (2nd order low-pass 0.1 Hz Butterworth filter) and a linear slope was removed from the spectrum spanning 100–400 Hz, which was computed from the average power within the 100–110 Hz and 390–400 Hz intervals. This procedure is illustrated in Fig. 2. A linear slope based on raw power more adequately captured background activity in this frequency range than a slope based on log-transformed power. The result of this automatic peak detection was carefully examined by eye. In case a peak was identified that was deemed not representative of the beta or HFO range, e.g. due to the 1/f-like shape of the spectrum for low frequencies, the second or third largest maximum was designated as the peak for that recording site. This mainly affected sites that were not selected as having maximum beta or HFO power (beta: 4% of selected versus 15% of unselected sites; HFO: 0% of selected versus 25% of unselected sites). Cases in which no clear peak could be identified in the beta (*n* = 1) or HFO range (*n* = 4) in any of the recording sites were excluded from the data, leaving 22 nuclei from 13 patients. Typically, a single peak dominated within the beta and HFO range, while multiple peaks were rarely observed.

In addition to spectral power, we looked at beta-HFO phase-amplitude coupling and identified the site with the largest PAC per nucleus. Here we opted for the average value across all beta and HFO frequency combinations within the spectrum rather than the peak value to reduce the influence of noisy outliers and to account for the spatial extent of PAC across frequencies. For this we computed PAC values using a general linear model (Penny et al., 2008, van Wijk et al., 2015). Firstly, we obtained low- and high-frequency components of the LFP signals by bandpass-filtering around centre frequencies between 5 and 35 Hz with 1 Hz steps (filter bandwidth ± 1 Hz), and between 150 and 400 Hz in steps of 2 Hz (filter bandwidth ± 35 Hz). Subsequently we extracted the instantaneous phase for each low-frequency component via *θ*_*x*_ = mod(angle(hilbert(*x*)), 2*π*), and amplitude of each high-frequency component via *a*_*y*_ = abs(hilbert(*y*)). We then formulated a general linear model as: *a*_*y*_ = *β*_1_ sin(*θ*_*x*_) + *β*_2_ cos(*θ*_*x*_) for each combination of low frequency components *x* and high frequency components *y*. The *β*-coefficients were estimated via least squares and combined to yield a single PAC value per frequency combination: $\;r=\sqrt{\beta_{1}^{2}+\beta_{2}^{2}}$. PAC values were averaged within the beta (13–30 Hz) and HFO (150–400 Hz) frequency intervals and the site with the largest average was identified. In order to assess the significance of PAC in individual spectra, we divided the continuous time series into 3.5 s non-overlapping epochs and repeated the estimation of the *β*-coefficients for each epoch. We then used parametric statistics to compare the distribution of *β*-coefficients across epochs against zero. See van Wijk et al. (2015) for more details of this method. A Bonferroni correction was applied to correct for the number of frequency combinations within the PAC spectrum (α = 0.05 / 4410).

The number of nuclei was counted in which the sites with the largest values for the different measures coincided, and was expressed as a percentage out of all nuclei. The expected number of observed nuclei due to chance was computed by assuming an a priori uniform probability across sites for each measure independently. A binomial test was then performed to compute the probability of finding the experimentally observed number of nuclei. In addition, we looked at the spatial distribution of locations with maximum beta/HFO power and PAC. This was expressed relative to the surgical target point as well as relative to the fixation site, in order to predict the distribution across contact pairs after implantation. We also tested for differences in MNI coordinates between sites with maximum power or PAC using paired-samples *t*-tests.

## 3 Results

**Fig. 3:**
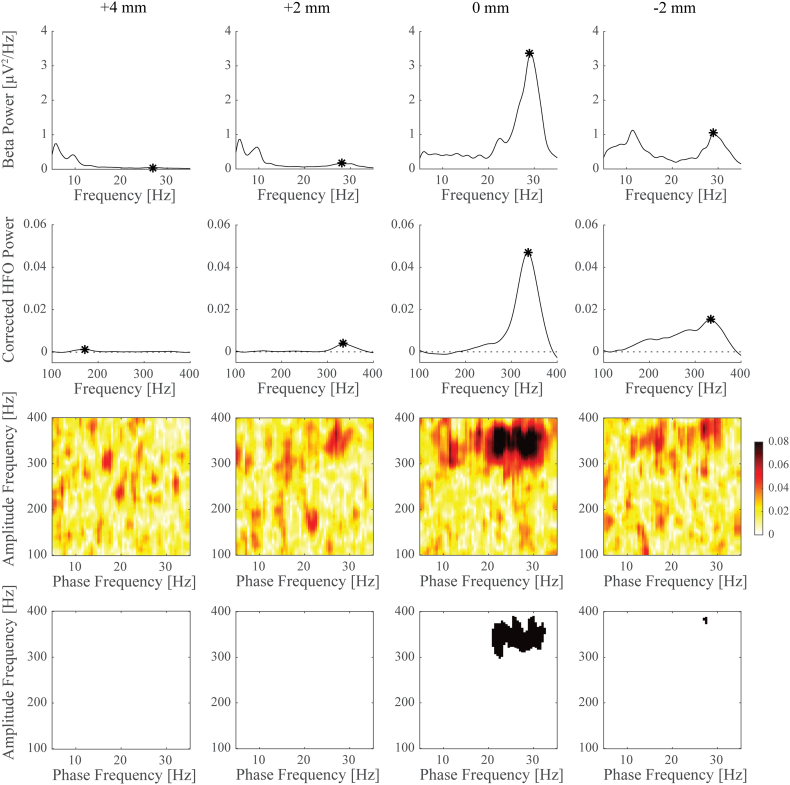
Power spectral densities and phase-amplitude coupling for different recordings sites in an example nucleus. Top row shows spectral power for frequencies around the beta band, second row shows corrected power (low-pass filtered and detrended) for the HFO frequency range. Peaks selected for comparison across sites are marked with an asterisk. PAC between beta and HFO frequencies is shown in the third row where warmer colours indicate stronger coupling. The corresponding panels in the fourth row indicate for which frequency combinations PAC was found to be significant (in black) after applying a Bonferroni correction. In this example, largest beta power, HFO power, and PAC were all found at the same recording site.

Fig. 3 illustrates an example of estimated power and PAC spectra for all recording sites in a single nucleus. Here, the largest beta band power was observed at the same recoding site as the maximum HFO power. This also coincided with the largest average beta-HFO PAC, which was found to be significant in this case. We found significant PAC in at least one of the recording sites in 15 nuclei (68% of cases). Table 1 lists for all individual nuclei the peak frequencies of identified maximum power and PAC and at which recording site they were found.

**Table 1:** Individual peak frequencies of identified recording sites with largest beta/HFO power and PAC. The frequency resolution for spectral power was 0.2 Hz. Nuclei for which PAC was found to be significant are indicated with an asterisk. Although we averaged PAC values across the spectrum in our main analyses, peak frequencies are listed here for comparison with spectral peak frequencies.

Case	Left hemisphere						Right hemisphere					
	Beta peak frequency [Hz]	Recording site [mm]	HFO peak frequency [Hz]	Recording site [mm]	PAC peak frequency [Hz]	Recording site [mm]	Beta peak frequency [Hz]	Recording site [mm]	HFO peak frequency [Hz]	Recording site [mm]	PAC peak frequency [Hz]	Recording site [mm]
1							29.0	0	336.0	0	23/342*	0
2							17.2	+ 4	252.8	+ 4	21/218	0
3	16.8	0	225.6	+ 4	17/250*	+ 4	16.8	0	231.8	+ 4	18/256*	+ 4
4	13.4	+ 4	241.8	+ 2	15/274*	+ 4	13.4	− 2	244.2	+ 4	15/262*	+ 4
5	19.4	+ 4	227.8	+ 4	19/234*	+ 4	20.4	+ 4	231.2	+ 4	21/264*	+ 4
6	16.0	+ 4	238.4	+ 4	16/254*	+ 4	15.6	+ 4	236.6	+ 4	16/256*	+ 4
7	14.6	− 2	234.2	+ 2	20/352	+ 2	20.4	0	224.6	+ 2	30/196	− 4
8	18.8	0	246.4	0	27/266*	0	19.8	0	251.0	0	18/272*	0
9							16.4	− 2	224.2	+ 4	16/224	− 2
10	25.0	0	248.6	+ 2	17/276	+ 2	25.2	+ 2	240.2	+ 4	22/312	0
11	17.4	+ 2	236.2	+ 2	18/252*	+ 2						
12	21.2	+ 2	244.0	+ 2	23/274*	+ 2	20.6	0	339.8	− 2	16/368*	0
13	14.2	− 2	220.8	0	19/328	+ 4	23.0	+ 2	270.0	+ 2	17/374*	+ 2

**Fig. 4:**
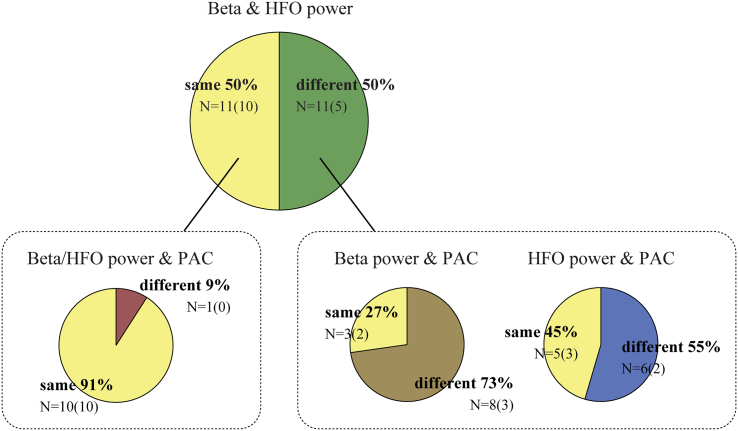
Overlap in identified recordings sites with largest beta and HFO power. The number of observed nuclei in which the largest power and/or PAC was observed in the same or in a different recording site is indicated, and in addition expressed as a percentage of the total number of cases. Observations were split in nuclei with and without overlap in beta and HFO power and further examined for overlap with largest PAC. Largest PAC was highly likely to be found at the same recording site if there was an overlap between beta and HFO power. For cases without overlap in beta and HFO power, PAC tended to occur more often at the site of largest HFO power than beta power. The number of nuclei in which PAC was found to be significant after applying a Bonferroni correction is indicated in brackets.

When considering all nuclei, we found an overlap in the electrode track depth of maximum beta and HFO power in 50% of cases. The probability of observing the largest value of two measures at the same recording site due to chance alone equalled 0.247, meaning that the chance of randomly observing 11 out of 22 cases was *p* = 0.009. The overlap between beta power and PAC was found to be lower (59%, *p* < 0.001) than for HFO power and PAC (68%, *p* < 0.001). In 45% of cases largest beta power, HFO power, and PAC occurred at the same recording site, which was considerably higher than the number of cases expected by chance (*p* < 0.001). Remarkably, 91% of cases where largest beta and HFO power was found at the same recording site also had the largest PAC at this location, and PAC was found to be significant in all of them. Looking only at cases where largest beta and HFO power were identified at different locations, largest PAC was identified at the same site as beta power in 27% of cases (*p* = 0.502) and in 45% (*p* = 0.094) of cases at the same site as HFO power. These results are visualized in Fig. 4.

**Fig. 5:**
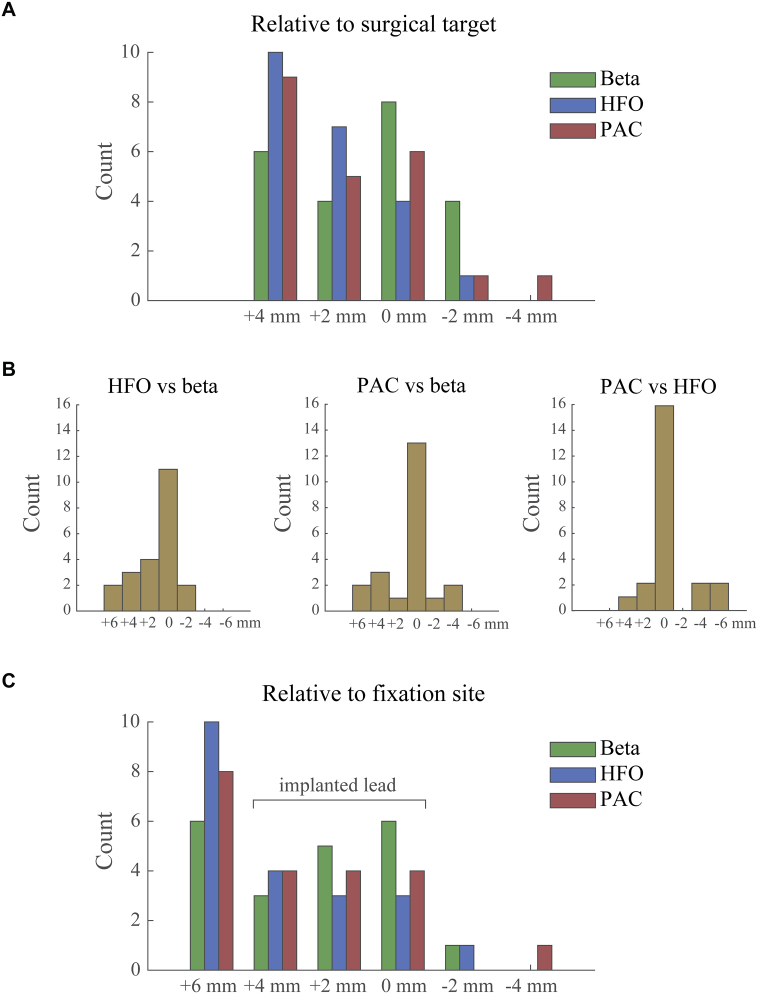
Spatial distribution of largest beta/HFO power and PAC across recording sites. All plots show histograms with the number of observations. Panel A depicts the distribution of recording sites relative to the surgical target point. The relative locations of largest power and PAC within nuclei are shown in panel B. Although largest beta and HFO power occurred at the same recording site in half of the nuclei, HFO tended to peak at slightly more superior sites for the other nuclei. Panel C depicts the distribution of recording sites relative to the fixation site of contact C0. The range spanned by the implanted lead is indicated in the Figure. This reveals that several sites with largest power or PAC are not covered by the lead after implantation. In all cases, positive distances indicate more superior locations. For nuclei with 5 recording sites available the relative distance between sites with maximum power or PAC did not exceed 6 mm. The nucleus where contact C0 was fixated at − 1 mm was discarded from the relative to fixation site histogram.

An interesting pattern emerged when looking at the spatial distribution of beta/HFO power and PAC (Fig. 5A). While largest beta power was most likely to occur at the surgical target point site, largest HFO was detected most often at the first recording site, 4 mm above the target point, and increasingly less often for subsequent sites. This distribution however does not convey the relative location between maximum sites within a nucleus. We therefore also express the sites with largest power or PAC relative to one another in Fig. 5B. This confirmed the detection of largest HFO at more superior sites relative to beta power. A similar tendency could be observed for the relative distribution of beta power and PAC, but a more symmetric distribution was found for PAC relative to HFO power. In order to determine which contacts are expected to cover the locations with largest power or PAC after lead implantation, we show in Fig. 5C the spatial distribution with respect to the site where the lowermost contact was eventually fixated (often 2 mm below the target point). This revealed that largest HFO is most likely to be found at 6 mm above the fixation site, which corresponds to a location not covered by the implanted lead as it would require a derivation between the most proximal contact and another one 2 mm above. Similarly, several sites with largest beta power are also not covered. The sites with largest power or PAC that are covered by the implanted lead are expected to be distributed approximately equally across contacts.

**Fig. 6:**
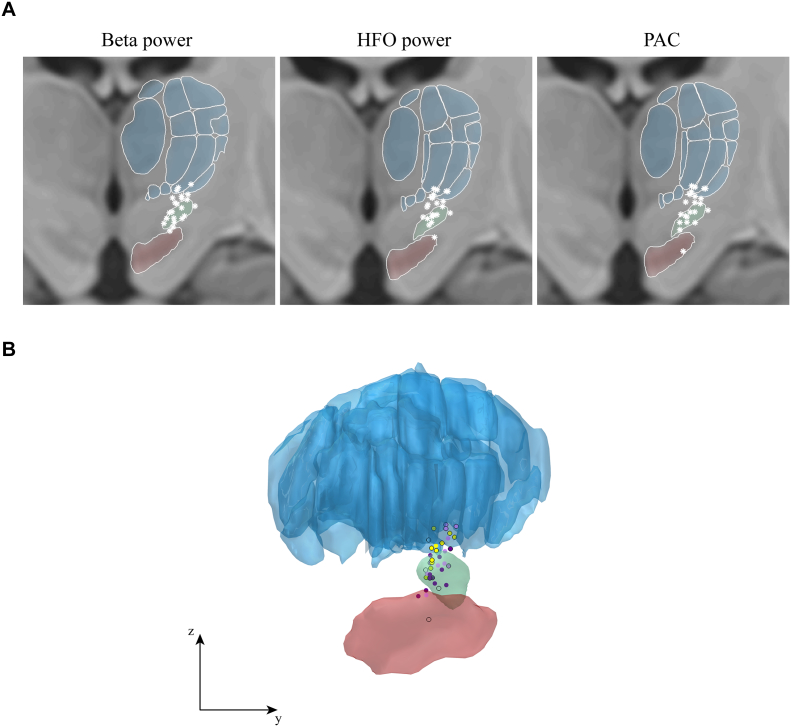
Locations of identified sites with largest power or PAC. Panel A shows the coronal plane for each measure separately. Each white star represents the finding of a single hemisphere. STN is outlined in green, thalamic structures in blue, and the substantia nigra in red. Locations with largest beta power significantly different from locations with largest HFO power in *x*-, *y*-, and *z*-direction. Locations with largest PAC did not significantly differ from either beta or HFO power. Panel B shows all peak locations from a lateral view. Sites where largest beta power was found are shown in dark purple, sites with largest HFO in light purple, and sites where largest beta and HFO coincided are indicated in yellow. Black circular outlines represent the sites where largest PAC was found.

Visualization of the 3D coordinates in the coronal plane made this point even more clear (Fig. 6A). While largest beta power was found at various depths within the STN, largest HFO power was detected more often in the upper part and above. Indeed, coordinates of the sites with largest beta power (average MNI coordinates = 12.12, – 14.01, − 5.64 mm) were significantly different from the coordinates of largest HFO power (12.46, – 13.56, − 4.32 mm) in *x*-direction (*t*(21) = − 2.47, *p* = 0.022), *y*-direction (*t*(21) = − 2.38, *p* = 0.027) and *z*-direction (*t*(21) = − 2.72, *p* = 0.013). The coordinates for the sites with largest PAC (12.31, – 13.74, − 4.86 mm) did not differ from the sites with largest beta or HFO power in any of the directions (*p* > 0.17). All peak locations are presented in a single figure in panel 6B.

Despite more recordings with significant PAC for nuclei in which beta and HFO power were found at the same site compared to nuclei without overlapping sites, the difference in average PAC did not reach significance (independent samples *t*-test, *t*(20) = 0.97, *p* = 0.345). Nor was there a significant difference in largest beta power or HFO power between nuclei with and without overlapping beta and HFO sites (*t*(20) = 0.89, *p* = 0.382; *t*(20) = − 0.16, *p* = 0.876). Pre-operative contralateral hemibody bradykinesia/rigidity UPDRS scores were similar as well (*t*(20) = − 0.87, *p* = 0.393). Hemibody bradykinesia/rigidity UPDRS scores were also not found to be correlated across nuclei with beta peak value (*r* = 0.27, *p* = 0.222), HFO peak value (*r* = 0.12, *p* = 0.601), average PAC (*r* = 0.31, *p* = 0.167), beta peak frequency (*r* = − 0.07, *p* = 0.759) or HFO peak frequency (*r* = − 0.16, *p* = 0.473). Finally, we looked at correlations between peak values for beta power, HFO power, and average PAC across the 10 nuclei for which these features were localized to the same recording site. Only beta power and PAC were found to be significantly correlated (*r* = 0.77, *p* = 0.009). No significant correlation was found between beta and HFO power (*r* = − 0.22, *p* = 0.533), nor between HFO power and PAC (*r* = 0.08, *p* = 0.823).

## 4 Discussion

Using intraoperative bipolar recordings of LFPs through DBS electrode contacts, we identified the relative location of strongest beta band oscillations, high-frequency oscillations, and beta-HFO phase-amplitude coupling within the region of the subthalamic nucleus of Parkinson's disease patients. Largest beta, HFO and PAC spectral peaks were found at the same recording site in half of the nuclei investigated. In nuclei without overlap in beta and HFO power, HFO tended to occur at a more superior location than beta power. In these cases, the site with strongest PAC was slightly more likely to be found at the site with largest HFO, although numbers were too small to confirm a statistical relation. These findings contribute towards the neurophysiological interpretation of HFO and beta-HFO PAC, and may serve as a foundation for studies aiming to improve DBS targeting.

Previous studies have investigated the spatial distribution of beta power (Kühn et al., 2005, Steigerwald et al., 2008, Zaidel et al., 2010, Seifried et al., 2012), HFO power (Wang et al., 2014) and beta-HFO PAC (Yang et al., 2014) in the subthalamic nucleus. All demonstrate largest power/PAC values in the dorsolateral STN, around 2 mm below its superior border. From these independent studies, it is unclear what relative source locations are underlying these various data features. Wang et al. (2014) identified the location of HFO to be deeper and more anterior compared to the DBS target site. Given the correspondence between the occurrence of beta oscillations and the location with largest clinical response (Chen et al., 2006, Yoshida et al., 2010, Zaidel et al., 2010), this suggest that HFO might be located more ventral than beta oscillations. The present study is the first to directly compare the relative location of beta and HFO power and beta-HFO PAC within the same nucleus. Our findings indicate the same or even a more superior location of HFO compared to beta power.

While most studies on oscillatory activity in Parkinson's disease have focused on the beta band, relatively little is known about the neuronal origins of HFO. It is thought to reflect activity independent from neural spiking recorded in the signal (Wang et al., 2014, Yang et al., 2014). Like gamma band activity (~ 60–90 Hz), the amplitude of HFO increases with movement (Foffani et al., 2003, López-Azcárate et al., 2010, Litvak et al., 2012, Tan et al., 2013). Several studies have noted a shift in peak frequency upwards after administration of dopaminergic medication but report conflicting findings regarding the effect on amplitude, as well as on the relation between HFO amplitude and UPDRS scores (López-Azcárate et al., 2010, Özkurt et al., 2011, Wang et al., 2014, van Wijk et al., 2016). It also remains to be established whether HFO directly relate to the bursting activity that is associated with Parkinson's disease, where intraburst discharge rates might fall within the HFO range (Wichmann and Soares, 2005, Gale et al., 2009, Sharott et al., 2014).

The presence of a spectral peak within the HFO frequency range seems critical for significant PAC to occur. Cases with significant PAC all had the largest HFO power peak present at the same recording site or at least a discernible subpeak. The 4 nuclei we excluded from analysis based on the lack of a clear peak within the HFO frequency range also failed to show significant PAC. There is a possibility that in these cases electrodes were positioned in a suboptimal location for detecting HFO or that HFO and PAC are more sensitive to stun effects than beta activity. While high beta and HFO power would improve the detectability of PAC because of more reliable phase and amplitude estimates, PAC did not blindly follow the site with largest beta power and was uncorrelated with HFO peak amplitude. Also beta and HFO peak amplitudes were uncorrelated (*r* = 0.17; *p* = 0.093), suggesting that these peaks did not merely occur by picking up more signal overall at certain sites. Our observation that PAC tended to follow the site with a clear HFO peak is in line with the study by Meloni et al. (2015) who compared the occurrence of beta-HFO PAC between different contact pairs along the electrode lead.

Considering that the volume of the STN is roughly 6 × 8 × 9 mm (Mavridis et al., 2013), it is almost inevitable that some of the implanted contacts are outside the STN. The spatial range covered by our intraoperative recordings made during the electrode descent was even larger and from the 3D visualizations it becomes clear that it is possible that some of the identified peak locations might reflect thalamic activity. This especially applies to the sites with largest HFO power, which were found significantly more superior than the sites with largest beta power. Interestingly, a recent intraoperative study with combined LFP and single unit recordings also observed HFO above the STN border (Telkes et al., 2016).

A limitation in the study resides in problems inherent to DBS imaging (Horn and Kühn, 2015). The application of neuroimaging methods to localize DBS electrodes comes with issues that we tried to minimize but that cannot be fully overcome. For instance, the linear coregistration between post- and preoperative acquisitions does not account for brainshift that may happen when opening the skull during surgery. Having said that, we employ several steps to minimize CSF leak and brain shift during surgery (Petersen et al., 2010) that result in minimal shift of the STN (Hyam et al., 2015). In addition, to co-register localizations into standard space across patients, nonlinear warps were applied that can never be perfectly precise and introduce problems to the field of neuroimaging as a whole (e.g., Klein et al., 2009, Ashburner and Friston, 2011). To minimize bias introduced by these methodological limitations, our pipeline was specially designed for the task of DBS electrode localizations and cross-validated for both postoperative MRI and CT (Horn and Kühn, 2015). Moreover, the processing stream has been applied and validated in numerous studies that involved large cohorts of subjects (Horn et al., 2017a, Horn et al., 2017b, Horn et al., 2017c). Each of the critical steps (co-registration, nonlinear normalization and electrode localization) included a manual control step in which results were critically reviewed by an expert. Additionally, final results of the electrode localization were reviewed by an expert neurosurgeon to ensure consistency with the post-operative MRI image and the surgeon's report. Still, a gold-standard (such as postmortem histology) is lacking and the precision of the applied methods is not perfect. The root mean square error may only be assumed to be of the same order of magnitude of ~ 1.3 mm reported by Schönecker et al. (2009) who used a predecessor of our current localization pipeline.

Furthermore, the bipolar derivation used in our analyses allowed for a more accurate estimation of local electrophysiological activity (Marmor et al., 2017) but introduces uncertainty regarding the contribution of each of the two contacts to the signal, and therefore the exact location of the beta/HFO source. This is somewhat resolved by comparing amplitudes between adjacent recording sites. Unless both contacts are located within the generation zone, the bipolar pair with the largest amplitude can be assumed to be closest to the source. We did not observe a clear difference in spatial extent of beta and HFO peaks. Both peaks were often present across adjacent recording sites but diminished in amplitude. The systematic difference in location between beta and HFO peaks suggests that our findings are uninfluenced by these factors.

We noticed a striking correspondence for both beta and HFO peak frequencies between left and right nuclei of the same subject, which has been observed before (de Solages et al., 2010). Still we have treated left and right nuclei as independent samples in our analysis to increase our sample size. For the majority of cases both sides were included in the analysis, and subjects hence equally contributed to the results. The identified recording sites with largest power or PAC seems a bit more variable between hemispheres, as we observed 4 cases with identical sites for power and PAC in left and right nuclei and 6 cases with different sites.

The positive correlation between beta-HFO PAC and UPDRS scores for bradykinesia and rigidity has led studies to speculate that beta band oscillations constrain pro-kinetic HFO activities, therefore hampering movement (López-Azcárate et al., 2010, van Wijk et al., 2016). Given the strong co-variation between beta-HFO PAC and beta band power, this could mean that any causal relation between high levels of beta oscillations and motor impairments may be mediated via beta-HFO PAC (or at least the two are highly inter-related). If so, therapeutic interventions to reduce PAC or to stimulate HFO activity might both be successful.

At present, the mechanisms of action of DBS remain under debate. Both direct electrical effects of stimulation and more indirect effects on neurotransmitter release may play a role in reducing clinical symptoms, and stimulation acts on efferent as well as afferent projections (Herrington et al., 2015, Udupa and Chen, 2015). Some studies even found the most effective site for stimulation to be located just above the superior border, hence outside the STN (Lanotte et al., 2002, Voges et al., 2002). Unfortunately, we do not have measures of clinical outcome upon stimulation available for all recording sites, making a direct comparison between the presence of beta/HFO power or PAC and effect of stimulation difficult in the current study. It would be interesting to see whether DBS is more effective when stimulation is applied through electrodes from which HFO and beta-HFO PAC could be recorded. If so, these data features may provide additional guidance for surgical targeting, and may also be useful for optimizing closed-loop stimulation (Little et al., 2013), or for steering current with directional leads (Bour et al., 2015).

The presented work provides an exploratory study in this direction. The relatively coarse sampling with 2 mm steps is insufficient to draw conclusions about fine spatial scales, but our findings indicate that the generators of HFO are closely, but slightly more superiorly located to those of beta oscillations. There is even still the possibility of multiple generating sites for beta and HFO oscillations that are spatially close to another. Future work would therefore benefit from a more detailed topographic mapping to resolve this. New developments in imaging methods and visualization techniques will substantially aid this direction of research.

## Funding sources

This research was supported by the National Institute for Health Research University College London Hospitals Biomedical Research Centre. PB is supported by the Medical Research Council (MC_UU_12024) and the National Institute for Health Research Oxford Biomedical Research Centre. The Wellcome Trust Centre for Neuroimaging is supported by core funding from the Wellcome Trust 091593/Z/10/Z. The Unit of Functional Neurosurgery is supported by the Parkinson's Appeal UK, and the Monument Trust. AH received funding from Stiftung Charité, Berlin Institute of Health and Prof. Klaus Thiemann Foundation. The authors declare no competing financial interests.
